# Leadless pacing: a comprehensive review

**DOI:** 10.1093/eurheartj/ehaf119

**Published:** 2025-03-19

**Authors:** Shmaila Saleem-Talib, Crispijn P R Hoevenaars, Nadine Molitor, Vincent J van Driel, Jeroen van der Heijden, Alexander Breitenstein, Harry van Wessel, Mathijs S van Schie, Natasja M S de Groot, Hemanth Ramanna

**Affiliations:** Department of Cardiology, Haga Teaching Hospital, The Hague, The Netherlands; Department of Cardiology, Haga Teaching Hospital, The Hague, The Netherlands; Electrophysiology, Department of Cardiology, University Hospital Zurich, Zurich, Switzerland; Department of Cardiology, Haga Teaching Hospital, The Hague, The Netherlands; Department of Cardiology, Haga Teaching Hospital, The Hague, The Netherlands; Electrophysiology, Department of Cardiology, University Hospital Zurich, Zurich, Switzerland; Department of Cardiology, Haga Teaching Hospital, The Hague, The Netherlands; Department of Cardiology, Erasmus Medical Center, Rotterdam, The Netherlands; Department of Cardiology, Erasmus Medical Center, Rotterdam, The Netherlands; Department of Cardiology, Haga Teaching Hospital, The Hague, The Netherlands; University of Applied Sciences of The Hague, The Netherlands

**Keywords:** Leadless pacemaker, Micra, AVEIR, CRT, CSP

## Abstract

Over the past decade, leadless pacing has undergone a rapid evolution, resulting in multiple leadless pacemaker (LPM) devices that offer advancements such as atrioventricular synchronized pacing in VDD mode, atrial stimulation, dual-chamber pacing, and longer battery longevity. Studies comparing LPMs with transvenous pacemakers (TVPMs) show a lower rate of device-related complications with LPMs. In the near future, LPMs could be combined with other devices such as non-transvenous implantable cardioverter-defibrillators to provide anti-tachycardia pacing or bradycardia pacing. Future prospectives for leadless cardiac resynchronization therapy and leadless conduction system pacing are being investigated. As LPMs continue to improve, their applications are anticipated to expand further improving patient outcome, promising a bright future for leadless pacing. In this review, the past, present, and future of leadless pacing are discussed with a focus on cutting-edge implantation techniques, clinical outcomes, and modern advancements of LPMs.

## Graphical Abstract

**Graphical Abstract:**
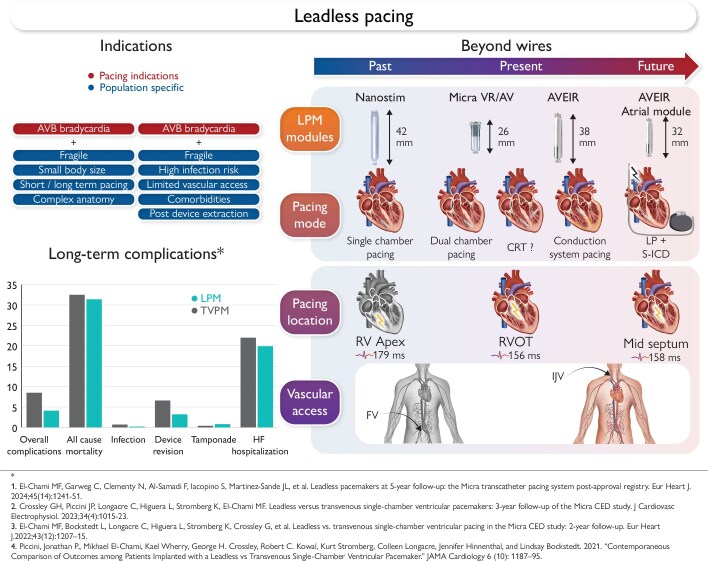
An overview of the past, present, and future of leadless pacing: comparison of different leadless pacemakers (LPMs) and their size in millimetre, with the Nanostim in the past, the Micra VR/AV and AVEIR with atrial module in the present. Furthermore, an overview of the different pacing modes: single-chamber pacing VVI/VDD mode, dual-chamber pacing DDD mode, cardiac resynchronization therapy (CRT) (it is not clear how this will be implemented, a LPM in the left ventricle is not desirable), conduction system pacing with a LPM in the interventricular septum activating the conduction system, and a LPM combined with a subcutaneous implantable cardioverter-defibrillator (S-ICD), which can deliver shock therapy. Different pacing locations with the right ventricular (RV) apex in the past, given the higher risk of pericardial effusion and the broader paced QRS complex. The vascular access with the femoral vein (FV) in the past, given more vascular complications, and the internal jugular vein (IJV) in the future with less vascular complications and easier access to the RV septum. Different pacing indications in the very young and the very old. Long-term complications in LPMs vs. transvenous pacemakers (TVPMs), showing lower overall complications, lower infection rate, lower device revision rate, and a lower heart failure (HF) hospitalization rate for LPMs. On the other hand, more cardiac perforations and a higher all-cause mortality rate for LPMs. AVB, atrioventricular block; RVOT, RV outflow tract.

## Introduction

The first permanent pacemaker was implanted in humans in 1958, marking a significant milestone in medical history.^1^ Despite the widespread success and use of transvenous pacemakers (TVPMs), both the leads and the subcutaneous pacemaker pocket have been identified as the ‘Achilles heel’ of these devices. Transvenous pacemakers still remain the gold standard, and their technology has rapidly evolved, with improvements in battery quality, device size, enhanced pacing algorithms, and increased lead durability. Despite these technological developments, TVPMs are not devoid of potential downsides and complications. In the long term, ∼10% of patients experience TVPM-related complications, with the vast majority of these complications being related to the transvenous lead or the subcutaneous pocket.^2–4^

To address these limitations, Dr Spickler conceptualized the first leadless pacemaker (LPM) in the early 1970s and tested its feasibility in dogs. Now 50 years later, his vision of an intra-cardiac self-contained LPM has become reality, with LPMs being implanted around the globe.^5–7^ The main advantage of the LPMs is the impressive reduction in complications by 50% compared with TVPMs, by preventing complications related to leads and pocket including infection, haematoma, pneumothorax, lead fracture, and lead dislocation. On the other hand, LPMs have more vascular complications and pericardial effusion compared with TVPMs.^8^

Since 2012, three different LPMs have been introduced: the Nanostim LPM (St. Jude Medical/Abbott, Chicago, IL, USA), the Micra transcatheter pacing system (Medtronic, Minneapolis, MN, USA), and the AVEIR LPM (Abbott). The first LPMs were limited to a selective group of patients, who only required ventricular pacing in VVI(R) mode. To enlarge this selective patient population, the next generation of LPMs was introduced offering atrioventricular (AV) synchronous ventricular pacing (Micra AV). Recently, the first dual-chamber LPM (AVEIR DR) is available offering all pacing modes including AAI(R), VVI(R), and DDD(R), but at a much higher cost compared with TVPMs. Leadless devices with resynchronization capacity are not available yet; hence, TVPMs remain superior to LPMs with respect to biventricular synchronized pacing.

In this review, we provide an overview of the indications, patient selection, and clinical outcomes of LPMs with particular focus on new insights.

## The past of leadless pacemaker device

### Nanostim

**Figure 1:**
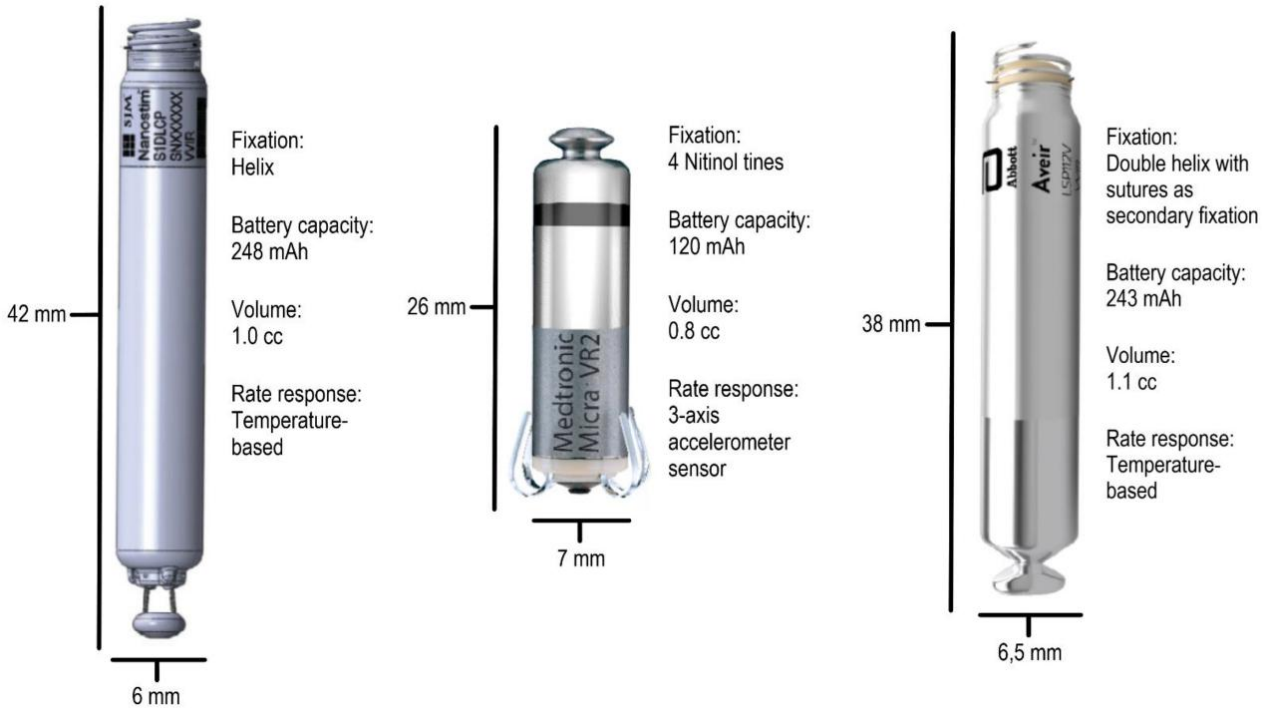
Comparison of the size and volume of the different leadless pacemakers. The Nanostim leadless pacemaker, the Micra leadless pacemaker, and the AVEIR leadless pacemaker with device characteristics

The first LPM was the Nanostim (St. Jude/Abbott). This 42 mm long intra-cardiac LPM with a volume of 1 cc and a screw-in-helix (*Figure 1*; *Graphical Abstract*) was implanted a total of 1423 times worldwide from December 2012 until March 2017.^9,10^ This was a single-chamber LPM, able to pace and sense in the ventricle and deliver rate response [VVI(R) mode]. The rate-modulating algorithm of the Nanostim depended on a temperature-based sensor. The LPM communication was utilized using conductive telemetry via five surface electrocardiogram electrodes to minimize battery drain during readouts.^11^ The screw-in-helix mechanism for fixation of the device within the ventricle allowed retrievability if necessary using a dedicated retrieval catheter, which could be used to snare the device and capture the distal cap followed by unscrewing the Nanostim through rotation in a counter-clockwise manner.

The LEADLESS trial was the first study to investigate efficacy and safety of the Nanostim; 33 patients were enrolled from 2012 to 2013 in 3 European and American centres. They reported an implant success rate of 97%.^7^ In two patients, serious adverse events however occurred. The first patient became haemodynamically unstable due to cardiac perforation requiring cardiothoracic surgery. This patient died 2 weeks after the LPM implantation due to ischaemic stroke. In the second patient, the Nanostim had to be retrieved from the left ventricle (LV), where it was accidentally implanted through a patent foramen ovale.^7,12^

The second larger LEADLESS II trial enrolled 526 patients from 56 centres in 3 countries with a follow-up of 6 months, confirming the high implantation success rate of 95.8%. Device-related serious adverse events occurred in 34 (6.5%) patients consisting of cardiac perforation (1.5%), device dislodgement (1.1%), device retrieval due to elevated pacing thresholds (0.8%), and vascular complications (1.2%) (*Table 1*).^13^

**Table 1:** Summary of data from the landmark trials

	Nanostim Leadless II^13^	Micra IDE^14^	Micra MAP EMEA^15^	Micra PAR^16^	AVEIR LEADLESS II (Phase 2)^17^
Number of participants (*n*)	526	725	928	1817	210
Implant success (%)	95.8	99.2	99.9	99.1	98
Mean follow-up (months)	6	4	9.7	6.8	14.4
Mean threshold at implantation (V)	0.82 (0.4 ms)	0.63 (0.24 ms)	0.61 (0.24 ms)	0.65 (0.24 ms)	0.84 (0.4 ms)
Mean threshold after 6 months (V)	0.53 (0.4 ms)	0.54 (0.24 ms)	0.61 (0.24 ms)	0.60 (0.24 ms)	0.65 (0.4 ms)
Total complication rate (%)	6.5	4.0	3.6	2.7	6.7
Pericardial effusion (%)	1.5	1.6	1.0	0.8	1.9
Pericardial effusion requiring surgical intervention (%)	0.2	0.3	0	0.1	1.0
Perforation leading to death (%)	0.2	0	0.1	0.1	0
Groin complications (%)	1.2	0.7	1.1	0.6	1
Device dislodgements (%)	1.1	0	0	0.1	1
Infection (%)	0	0	0.2	0.1	0

Summary of data from the landmark trials, including the LEADLESS II study (Nanostim), the Micra IDE study, the Micra Acute Performance European and Middle Eastern (MAP EMEA) registry, the Micra PAR, and the LEADLESS II Phase 2 trial (Aveir).

Although these studies reported excellent results in clinical practice, there were serious concerns regarding early battery depletion in 7 of 1423 (0.5%) implanted devices within 29–37 months post-implantation. This led to a recall of the Nanostim and subsequently all Nanostim LPMs were replaced and future implantations were cancelled.^10,18,19^

## The present of leadless pacemaker device

The first Micra VR LPM was implanted in Linz Austria December 2013, with currently more than 200 000 devices implanted since then.^20^ In comparison with the Nanostim, the Micra is shorter in length (25.9 vs. 42 mm) (*Figure 1*; *Graphical Abstract*). The smaller size and volume of the device body account for <2% of the right ventricle (RV) volume. Pre-clinical studies have shown the possibility of implanting up to 3 Micra LPMs simultaneously in the RV without device-to-device interactions.^21–25^ The Micra LPM consists of four Nitinol tines for myocardial fixation. The Micra VR is, as the Nanostim, a single-chamber LPM, capable of pacing and sensing in the RV [VVI(R)]. The rate-modulating algorithm is based on a three-axis accelerometer.^26,27^

The early performance of the Micra was studied in 140 patients from 11 different countries, with a successful implantation procedure in all patients. In more than half of the patients (59%), the device was successfully implanted at the first attempt. During the 3-month follow-up, 30 adverse events occurred in 26 patients, consisting of 9 cases of arrhythmias, 7 events at device placement site, 11 events at groin puncture site, and other including back pain in 3 patients.^20^

The second larger study to investigate the safety of the Micra was the Micra Investigational Device Exemption (IDE) study. In this study, 725 patients were enrolled from 56 centres with a 6-month follow-up period. In 99.2%, the implantation was successful, and the remaining unsuccessful implantations were attributed to four cardiac perforations, one patient with unsatisfactory pacing thresholds, and one patient with tortuous venous anatomy. During 6-month follow-up, 96.0% of the patients were free from major complications and 98.3% had an adequate pacing threshold.^14^ Likewise, in the Micra Acute Performance European and Middle Eastern (MAP EMEA) cohort with 928 patients included and in the Micra Post-Approval Registry (PAR) cohort, including 1817 patients, successful implantation rates equalled 99.9% and 99.1%, respectively.^15,16^ At 12 months, 98.1% and 97.0% of patients had adequate pacing threshold values. Major complications were experienced in 3.6% and 2.7% during 12-month follow-up (*Table 1*). Major complications included complications related to groin access, pacing issues, pacemaker syndrome, and cardiac perforation. With respect to the risk of thrombosis and embolism, the IDE and PAR studies reported on deep vein thrombosis (*n* = 1, 0.3%) and pulmonary embolism (*n* = 1, 0.1%). In the MAP EMEA study, vascular events were not reported. The use of antithrombotic therapy peri-interventional within these three studies was unfortunately not described. In another study, including 107 patients, only 1 case of thrombosis of the ipsilateral saphenous vein occurred in a female patient who was not anticoagulated.^28^ In 2016, a case report described thrombus formation at the tip of the Micra catheter during the implantation, and heparin was not administered during this procedure.^29^ Ever since, administering a bolus of heparin during the implantation procedure has been recommended. Importantly, deep vein thrombosis, pulmonary embolism, or thrombosis of the LPMs was not observed in our cohort of 449 patients of whom 133 did not receive any anticoagulants during follow-up.

In summary, the three large studies demonstrated excellent successful implantation rates of the Micra LPM and stable electrical parameters, although potentially fatal cardiac perforation (*Table 1*) and vascular complications related to access site remain a concern.

### Leadless pacemaker device with atrioventricular synchrony

In 2020, the US Food and Drug Administration approved the second generation of Micra LPM, the Micra AV (AV synchronous pacing). This technology enables AV-synchronized pacing based on the three-axis accelerometer algorithm, which allows sensing atrial mechanical activity resulting in dual-chamber sensing and AV-synchronized ventricular pacing (VDD mode). The Micra AV has the same length, volume, and design as the Micra VR LPM. The MARVEL 1 (*n* = 64; Micra Atrial tRacking using a Ventricular accelerometer) and MARVEL 2 (*n* = 75) studies report an AV synchrony (AVS; AVS was defined as a P-wave followed by a QRS complex within 300 ms) of 87.0% and 89.2%, respectively.^30,31^ However, in real life achieving AVS can be challenging in patients with heart rates >100 b.p.m. or diastolic dysfunction, leading to lack of appropriate sensing of the atrial contractions.^31^ Overall, Micra AV has led to a broader patient selection for LPMs and offers an option for the population with sinus rhythm (requiring not only VVI pacing), expanding the field of leadless pacing.

### Implantation location

The RV apical septum and RV apex were initially the preferred implantation locations for the Micra LPMs (*Graphical Abstract*). In the IDE registry, the main implantation site was the RV apex (66%) complicated by pericardial effusion in 1.6%.^14^ Afterwards, the RV septal area became the primary target in the PAR registry, which was associated with a four-fold reduction in pericardial effusion to a total of 0.4%^16^(*Table 1*). More recent studies demonstrate benefits of targeting higher RV septal implant locations, such as the RV mid-septum and RV outflow tract (RVOT). Indeed, Garweg *et al*.^32^ compared LPM implantation locations in 133 patients, RVOT position in 45 patients, RV mid-septum in 58, and RV apex in 30. While there were no differences in pacing threshold and R-wave sensing between these three sites, the narrowest paced QRS complex was obtained when pacing from the RVOT. These findings were confirmed by our own observations in a cohort of 82 patients, where the narrowest paced QRS complex was measured at the RV mid-septum and RV high septum compared with the RV apical septum.^33^ We could also confirm similar pacing thresholds, pacing impedance, and R-wave amplitudes for all three positions.

### Battery longevity

The Micra LPM has a lithium-hybrid CFx silver vanadium oxide battery. The manufacturer claims a battery longevity of ∼12 years in VVI mode with 100% ventricular pacing at optimal pacing threshold parameters. A real-world analysis with 7-year follow-up confirms this predicted longevity.^34^

### AVEIR

In 2022, the AVEIR LPM successor of the Nanostim was introduced. The AVEIR LPM has a design similar to the Nanostim LPM. It is shorter in length with 38 vs. 42 mm length compared with the Nanostim, but slightly larger in volume of 1.1 vs. 1.0 cm^3^, respectively. The AVEIR LPM has the same fixation mechanism as the Nanostim with a screw-in-helix system and the same temperature-based sensor for rate modulation.^35,36^ Similar to the Nanostim and the Micra VR, the AVEIR can sense and pace in the ventricle and offers rate response (VVIR mode). According to the manufacturer, the lithium carbon-monofluoride battery with improved capacity is claimed to have a battery longevity of 17.6 years, which however needs to be confirmed by future studies. An advantage of the AVEIR is the option to map the electrical local signal before screwing the LPM into the RV septum. The proximal docking button has also been improved in comparison with the Nanostim by attaching it directly to the LPM in order to prevent it from detaching during extraction.

The LEADLESS II Phase 2 study enrolled 210 patients who received an AVEIR LPM across 43 centres from 2020 until 2021. They report a 98% successful implantation rate and a mean follow-up of 14.4 months. Detailed description of the complications is provided in *Table 1*. The device electrical performance remained stable during 1-year follow-up.^17^

Two studies comparing the AVEIR and Micra LPMs (*n* = 67 and *n* = 50) reported a longer procedure and fluoroscopy duration during AVEIR implantations, which can be explained due to lack of experience with this new technology and the more extensive implantation procedure for the AVEIR LPMs. Both studies display a longer projected battery longevity of the AVEIR LPMs after 6 months compared with the Micra LPM. The electrical parameters remained stable in both LPMs.^37,38^

### Leadless vs. transvenous pacing

Several non-randomized studies have compared LPMs with TVPMs (*Table 2*). The Micra Coverage with Evidence Development (CED) study, with a long-term follow-up of 3 years, compared 6219 LPM with 10 212 TVPM patients. They revealed a 43% lower complication rate and 41% lower system revision rate in the leadless population. Additionally, incidences of device-related infections and hospitalizations due to heart failure (HF) were significantly lower in the leadless cohort. Despite the lower complication rate, the adjusted 3-year all-cause mortality rate was similar for both the LPM and TVPM population (*Graphical Abstract*).^39^

**Table 2:** Summary of the data of studies comparing leadless pacemakers with transvenous pacemakers

	Micra CED^39^	Micra PAR^40^	Garweg *et al.*,^41^ LVEF and TR	Boveda *et al.*,^42^ high-risk cohorts	Shtembari *et al.*,^43^ meta-analysis
Follow-up (years)	3	3	1	2	
Participants LPM (*n*)	6.219	1.809	27	9.858	8.340
Participants TVPM (*n*)	10.212	2.667	24	12.157	15.008
Complication rate (LPM vs. TVPM)	4.9% (LPM) vs. 7.1% (TVPM)	4.1% (LPM) vs. 8.5% (TVPM)			4.6% (LPM) vs. 7.3% (TVPM)
System revision rate (LPM vs. TVPM)	3.6% (LPM) vs. 6.0% (TVPM)	3.2% (LPM) vs. 6.6% (TVPM)			2.7% (LPM) vs. 4.8% (TVPM)
Mortality	Similar				Similar
Conclusion	Significantly fewer complications, reinterventions, HF hospitalizations, and infections in LPM cohort compared with TVPM cohort	Significantly fewer complications and system revisions in LPM cohort compared with TVPM cohort	No significant difference in LVEF decay and lower TR severity in LPM group compared with TVPM cohort	Fewer complications and revisions in high-risk LPM sub-analyses (malignancies, diabetes, TVD, and COPD)	Fewer reinterventions, device dislodgements, pneumothoraxes, and overall complications in LPM cohort. More PE in LPM cohort

Summary of the data of studies comparing LPM cohorts with transvenous pacemaker cohorts, including the Micra CED Study and the Micra PAR. Garweg *et al.*^41^ examined the LVEF and TR in the LPM cohort vs. the TVPM cohort. Boveda *et al.*^42^ performed sub-analyses of the high-risk cohorts from the Micra CED cohort.

TR, tricuspid regurgitation; TVPM, transvenous pacemaker; PE, pericardial effusion.

Recently, El-Chami *et al*.^40^ published the 5-year follow-up of the Micra PAR consisting of 1809 LPM patients and comparing them with a reference dataset of 2667 TVPM patients. After 3 years of follow-up, they reported a system revision rate of 3.2% in the LPM compared with 6.6% in the TVPM population (*P* < .001). The investigators attributed this difference to less device-related infections and less device upgrades in the LPM cohort. During longer follow-up (60 months), revision of the LPM was necessary in 4.9% of the population. Including device upgrades to either dual-chamber TVPMs or to CRT pacemakers.

These findings between LPMs and TVPMs are further supported by a meta-analysis by Shtembari *et al*.^43^ They demonstrated a 42% lower odds of complications in the LPM (*n* = 8340) vs. TVPM cohort (*n* = 15 008) and a 46% lower odds of reintervention in the LPM cohort, while no significant difference in mortality was reported (*Graphical Abstract*). Another meta-analysis by Ngo *et al*.^8^ comprising of five studies comparing Micra LPMs (*n* = 1030) with TVPMs (*n* = 2959) showed a 51% lower odds of complications at 1-year follow-up for the LPM vs. the TVPM cohort. The lower risk of complications plays an incremental role in the elderly and more vulnerable population. Boveda *et al*.^42^ summarized in a subgroup analysis of 9858 patients with a LPM that this subset of patients benefit from leadless pacing compared with transvenous pacing. In the subgroups with malignancies, diabetes, tricuspid valve disease (TVD), and chronic obstructive pulmonary disease (COPD), significantly fewer complications were reported in the LPM cohort compared with the TVPM cohort. Additionally, the LPM subgroup with diabetes, TVD, and COPD required significantly fewer reinterventions compared with TVPM patients in the same subgroups. These findings indicate that patients who are at higher risk of pacemaker-related complications benefit from LPMs (*Graphical Abstract*).

### Pericardial effusion

Although the incidence of complications is low in LPM populations, the rate of serious complications such as cardiac perforation remains higher, compared with TVPM populations. In the LEADLESS II (Nanostim) study, 8 of 526 patients (1.5%) had a perforation, including one fatal cardiac perforation.^13^ A comparable complication rate was observed in the LEADLESS II (Phase 2) study, in which 4 of 210 patients (1.9%) had a perforation, fortunately none was fatal.^17^

In the Micra IDE study, perforations occurred in 1.8% of the patients and deaths were not reported.^14^ The prevalence of perforations was further reduced by a four-fold to 0.8% in the Micra PAR study.^16^

Reduction in perforation was attributed to a learning curve, adjustments in the implantation recommendations, and potentially the preference for RV septal LPM implantation instead of RV apical implantation.

Despite these safety measures, the risk of cardiac perforation remains a concern during LPM implantation. We therefore recommend implanting LPMs only in centres with backup cardiothoracic surgery on site, since 27.3% of leadless pacemaker (LMP)-related perforations required sternotomy.^44,45^

### Tricuspid valve regurgitation

An increase in tricuspid valve regurgitation (TVR) has been a concern with TVPMs, since the RV lead passes through the tricuspid valve, potentially interfering with its function. Garweg *et al*.^41^ studied 27 patients with Micra LPM and 24 patients with TVPMs. After 12 months of follow-up, there was no significant difference in the decline in LV ejection fraction (LVEF) between groups. However, the severity of TVR was significantly lower in the Micra group.

A recent review paper on TVD associated with cardiac implantable electronic devices (CIEDs) described no significant difference in TVR comparing 53 patients with LPMs and 53 age- and sex-matched controls with a dual-chamber TVPM at 12 months.^46,47^

Additionally, in a meta-analysis including 297 patients with a LPM, there was no increase found in TVR after a median follow-up of 12.1 months.^48^ We hypothesize that, due to the absence of a pacemaker lead crossing the tricuspid valve, LPMs are less likely to impair valve function.

### Leadless vs. transvenous pacing difference in implantation techniques

In several countries, including the Netherlands, device implanting cardiologists are not always electrophysiologists. Electrophysiologist performing catheter ablation procedures are more experienced in femoral vein punctures and guidance of steerable large bore sheets in the right atrium (RA) and RV. Hence, LPM implantation by electrophysiologists may be preferable.

### Leadless vs. transvenous patient selection

Over time, more experience has been gained on patient selection for LPMs. Leadless pacemakers would be a suitable option for most patients requiring single-chamber pacing. Although most implanting cardiologists are restricted in various countries due to the higher cost of LPMs. Considering the higher costs, patients who will benefit more from LPMs than TVPMs (*Table 3*) should be carefully selected. Leadless pacemakers are superior to TVPMs in reduction of complications such as infections, haematoma, pneumothorax, and lead fracture or dislocation. Therefore, patients who have a higher risk of infection or bleeding, such as patients who are immunocompromised or have comorbidities, diabetes, renal insufficiency/dialysis, liver failure, and frailty, are likely to benefit more from LPMs. Another patient population are those with limited vascular access. Similarly, patients who underwent tricuspid valve surgery would benefit from avoiding a lead crossing the tricuspid valve apparatus and would be eligible for a LPM. Furthermore, patients not requiring frequent pacing, such as patients with occasional cardio-inhibitory syncope or transient AV block, would benefit more from a LPM. Furthermore very cachectic, frail patients with a higher risk of device pocket issues would therefore be eligible for LPMs. For some patients, their profession or performing contact sports would be a reason to prefer a LPM. However, patients who are very active and would require AV synchronous pacing above a heart rate of 100 b.p.m.; a Micra AV might not be the most optimal option. A DDD-LPM could be a better alternative, although given the substantial higher costs of this system, it would only be advised in the population at high risk for infections. Patients requiring biventricular synchronized pacing are not eligible for a LPM (*Table 3*).

**Table 3:** **Patient characteristics favouring leadless pacemaker or transvenous pacemaker implantation** ^94^

Patient characteristic	LPMs	TVPMs
Vascular		
Limited venous access Dialysis shunt	+ +	+/− +/−
Infection risk		
DM Renal disease Immunocompromised Frailty Previous (CIED) infection)	+ + + + +	+/− +/− +/− +/− +/−
HF		
TVR Tricuspid valve replacement with biological valve Mechanical tricuspid valve Moderate-to-severe LV dysfunction with ≥20% RV pacing Moderate-to-severe LV dysfunction with <20% RV pacing Resynchronization therapy	+ + − − +/− −	+/− − − + + +
Other		
Cosmetic or occupational reasons Atrial pacing AV-synchronized pacing	+ +/− +/−	+/− + +

DM, diabetes mellitus; +, recommended; +/−, may be used; −, should not be used.

### Jugular vs. femoral approach

**Figure 2:**
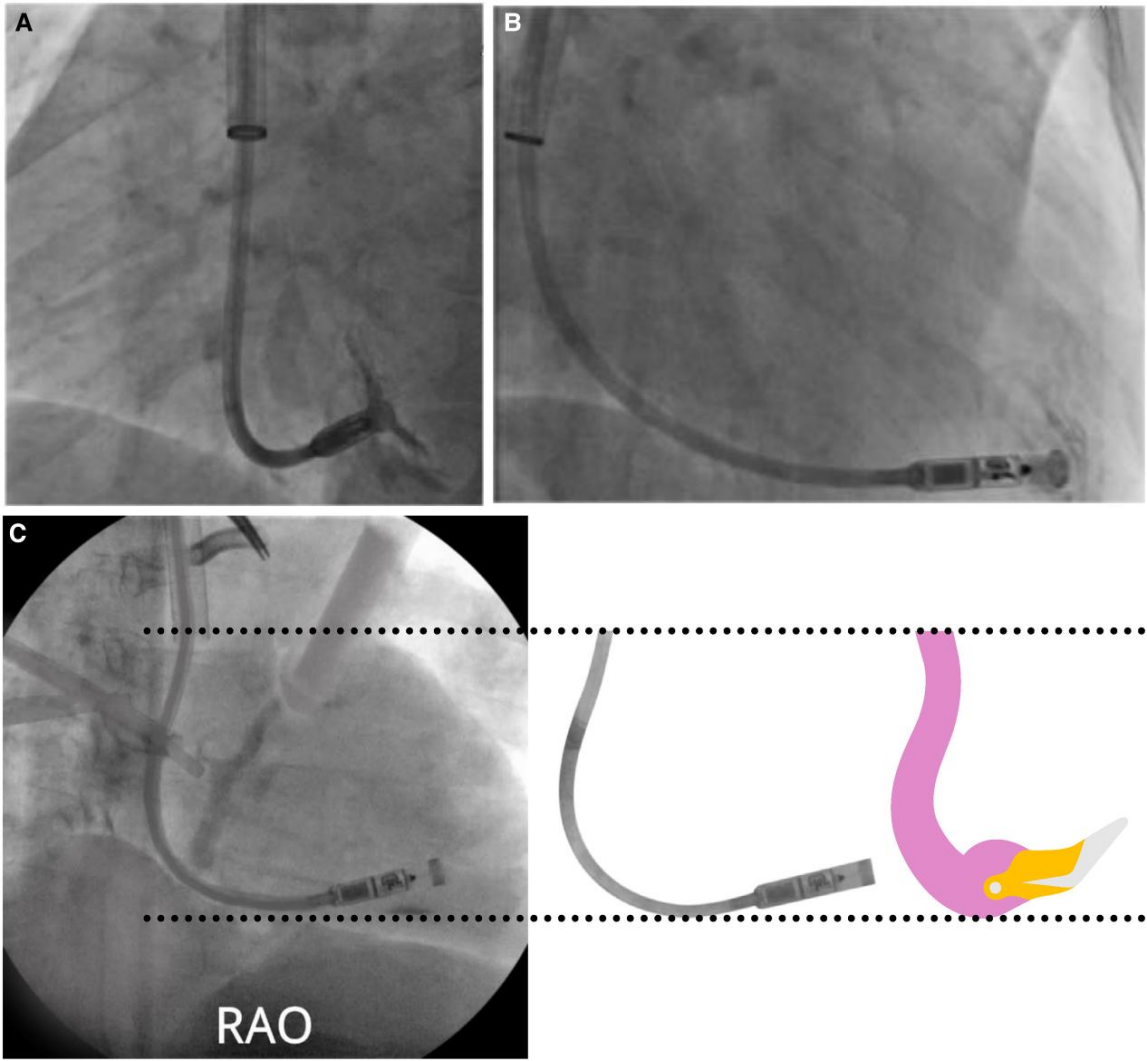
(*A*) Left anterior fluoroscopic image of the device positioned on the apical septum in the right ventricle with contrast injection approached through the internal jugular vein. (*B*) Right anterior fluoroscopic image of the device positioned on the apical septum in the right ventricle with contrast injection approached through the internal jugular vein. (*C*) Depiction of the flamingo neck confirming the right amount of push, with the jugular approach the force applied is transmitted 1:1

The common implantation technique for a LPM is through the femoral vein, although several studies and case reports have explored an alternative implantation route through the internal jugular (IJV) (*Figure 2*). Kolek *et al*.^49^ reported a safe implantation through the IJV in a 72-year-old patient, with a contraindication for LPM implantation through the femoral vein due to an inferior vena cava (IVC) filter. Following this pioneering method, our group further developed the jugular approach for LPM implantation in 82 patients with no access site related complications. Access site complications are often seen in the femoral approach for LPM implantations^33^ (*Graphical Abstract*). Moreover, several case reports have reported a jugular approach for the AVEIR LPM implantation, which is particularly noteworthy as the size of the AVEIR is ∼50% larger than the Micra LPM. The jugular approach was chosen, because of an unsuccessful femoral delivery due to a small RA, difficult angulation, or anatomical constraints.^50,51^

The main advantage of the jugular approach is the shorter route to the RA, bypassing the tortuous femoral veins, resulting in easier manipulation of the delivery tool and a less painful procedure for the patient. A recent multicentre study, which included 3D models of the heart by analysing cardiac computed tomography (CT) scans in small Japanese patients and comparing them with CT scans of larger American patients, demonstrated the access route from the IVC to the RV, showing a 38–40% larger angle to the tricuspid valve annulus, compared with the angle from the superior vena cava (SVC) to the tricuspid valve annulus. The approach from the SVC towards the RV results in a more natural curve towards the RV septum. They also depicted the delivery sheath of the Micra LPM in the 3D model, showing the cup of the delivery sheath pointing towards the RV septum, when entering the RV through the SVC, whereas the cup of the delivery sheath points towards the RV lateral wall when entering the RV through the IVC. Furthermore, the anterior position of the SVC in the mediastinum enhances this natural curve, facilitating the implantation of LPMs in higher RV septal regions. These non-apical locations are preferred to reduce the risk of cardiac perforation and achieving a narrower paced QRS complex in higher RV septal sites.^32,52,53^ Another benefit of the jugular approach is that patients can be ambulated immediately, effectively reducing discomfort. Particularly for those with back and joint issues, which are common in the elderly population. Moreover, the jugular approach facilitates same day discharge.^54^ In a recent multicentre study, the first 100 jugular LPM implantation procedures were compared with 100 femoral LPM implantation procedures. Both procedures were safe and effective, although the jugular procedures were associated with a shorter procedure time, lower fluoroscopy duration, reduced complications rates, and increased patient comfort.^55^ Although the easier manipulation from the jugular approach also leads to more force transmission during implantation. In the femoral vein approach, only 11% of the total force is transmitted, and therefore, the catheter should have a gooseneck shape to reassure good contact with the myocardium. In the jugular vein approach, almost 100% of the force is transmitted, and hence, it is not recommend to advance the delivery tool forward to create a gooseneck shape of the tool, but rather a ‘Flamingo’ neck shape (*Figure 2*).

### Leadless pacing in the very young and old population

Whether LPMs cause problems specifically in the very old or very young population is unknown.^2,56^ Overall, study data indicate that LPM implantation is both effective and safe in the elderly, with similarly low complication rates compared with younger patients.^57,58^ However, Hofer *et al*. reported a significantly longer procedure and higher fluoroscopy time of LPM implantations in patients >80 years old.^59^ An explanation for this prolonged procedure time could be more tortuous femoral/iliac veins or a medical history with interventions resulting in a changed RV geometry and function in combination with multiple comorbidities. The jugular approach may present a more favourable approach in such patients, but further studies are warranted.

Looking at the very young, several studies have investigated implantation of LPMs in the paediatric population. Siddeek *et al*.^60^ were the first to examine Micra implantations in children from 2018 to 2020 (*n* = 7), including three successful implantations through the jugular vein, all 3 patients weighing <30 kg. Another international study by Shah *et al*.^61^ included 63 children. In this study, 32% of the patients had a congenital heart disease. The main pacing indications were AV block in 40 patients (63%) and sinus bradycardia in 21 patients (33%). Eight patients weighed <30 kg, with the youngest being 4 years old. In this juvenile cohort, LPMs appeared feasible with stable electrical parameters observed during follow-up. However, 10 complications (16%) occurred during and after implantation. Three were major complications requiring intervention, including one pericardial effusion that required pericardiocentesis and one LPM that could not be implanted due to lack of myocardial capture. The third patient developed high pacing thresholds 24 h after implantation, necessitating the extraction of the prior LPM and implantation of a new one. The remaining complications were mostly vascular complications. The relatively high number of complications can be partly explained by the vulnerability of this complex and low body size cohort (*Graphical Abstract*).

### Dual-chamber leadless pacemakers

For the majority of patients with sinus node and AV node disease, dual-chamber pacing is preferred^62^ (*Graphical Abstract*). Consequently, relying solely on a ventricular LPM proves insufficient in this population, as they require some degree of atrial pacing or AVS pacing. While the Micra AV LPM offers AV synchronous pacing, it is limited by the lack of atrial pacing and restricted in providing AVS pacing in higher heart rates. To overcome this challenge, an AVEIR atrial module LPM has been introduced, which offers both atrial pacing and can be combined with a ventricular LPM for dual-chamber leadless pacing^63^ (*Graphical Abstract*). Several studies examined the communication between these leadless atrial and ventricular LPMs *in vivo* and reported viable results.^64^

The first study with 300 patients, receiving this dual-chamber AVEIR LPM, reported a successful implantation in 98% of patients. AVS pacing could be achieved at least 70% of the time, although 97% of patients had AVS pacing, more than 95% of the time in different postures. It is advised to implant the atrial module at the base of the right atrial appendage, although peri-procedurally four patients had atrial LPM dislodgements due to inadequate fixation (1.3%) and a system revision during follow-up was required in eight patients due to dislodgements of the atrial LPMs (2.7%). Most patients (*n* = 271) remained free of complications, with the vast majority of complications arising in the initial 2 days post-implantation.^65^

Unlike previous LPM models limited to ventricular pacing, this innovation enables ventricular and atrial pacing offering a leap into the future.

## The future of leadless pacing

### Leadless conduction system pacing

Right ventricular pacing is associated with ventricular dyssynchrony, resulting in a higher incidence of atrial fibrillation, HF, and mortality.^66^ To overcome this, CRT devices were implemented worldwide.^67^

For one third of the patients, CRT did not improve LV function as anticipated, and they were categorized as non-responders to CRT. Even though CRT is still the gold standard according to the pacing guidelines,^62^ conduction system pacing (CSP) as a novel pacing modality may be an option for this population. In CSP, the RV pacing lead is screwed into the RV interventricular septum, thereby capturing the patient’s own conduction system, resulting in biventricular synchronized pacing (*Graphical Abstract*). This pacing lead can be implanted either at the bundle of His [His bundle pacing (HBP)] or at the left bundle branch (LBB) area [LBB area pacing (LBBAP)].^68^ Left bundle branch area pacing has become the preferred method over HBP due to the larger anatomical target site, shorter procedure time, and more stable electrical parameters.^69,70^ However, in some patients, CSP does not result in capturing the conduction system. At present, there is lack of data on stability of the CSP electrical parameters over time and how to manage extractions of CSP leads.

In regard to leadless CSP, no studies have been published. To achieve CSP, with current LPMs, smaller devices with adjusted fixation mechanisms are required. These devices are in development.

### Leadless pacemaker and subcutaneous implantable cardioverter-defibrillator

The subcutaneous implantable cardioverter-defibrillator (S-ICD) is an established alternative to the transvenous ICD.^71^ Nevertheless, one of the shortcomings of the S-ICD is the lack of pacing for bradycardia or anti-tachycardia pacing (ATP). To address this shortcoming, the S-ICD has been combined with a LPM in several case reports and in pre-clinical animal studies^72–74^ (*Graphical Abstract*). A modular cardiac rhythm management system combining the S-ICD with a LPM in the RV in one communicating system has been introduced by Boston Scientific. Initial pre-clinical studies on animals with a follow-up of 18 months demonstrated a high rate of successful implantations and stable electrical parameters over time.^75,76^ Following this initial work, the prospective MODULAR ATP study was designed and recently published. In this system, the S-ICD (Emblem) is combined with a novel LPM (EMPOWER). A total of 293 patients were enrolled, and more than half of them completed the 6-month follow-up period. The percentage of patients without major complications was 97.5%, and 147 patients (97.4%) had pacing threshold values of ≤2.0 V. In patients with sustained monomorphic ventricular tachycardia, 61.3% episodes of arrhythmias were successfully terminated by ATP delivered by the LPM.^77^

### Leadless cardiac resynchronization therapy

The cornerstone of HF device treatment is CRT, offering significant clinical benefits.^78^ Nonetheless, these systems are not devoid of lead-related problems including lead fractures and device-related infections.^79^ Not to mention one third of patients failing to benefit from conventional CRT treatment (non-responders). Many studies have tried to overcome these problems; however, CRT still remains susceptible to complications.^80,81^

To provide an at least partial leadless solution for CRT, the WiSE-CRT system has been introduced, consisting of a device capable of wireless LV endocardial pacing, using a passive electrode implanted in the LV endocardial wall. Communication takes place through ultrasound waves from a transmitter located subcutaneous below the apex of the heart, which is in turn in contact with a transvenous system in the RA and RV.^82^ A systematic review and meta-analysis demonstrated the usefulness of this system for patients in whom conventional CRT fell short due to issues related to the LV lead.^83^ The original WiSE-CRT system is not completely leadless, since it has leads in the RA and RV. Nevertheless, methods to combine the Micra LPM and the WiSE-CRT system have been explored to create a total leadless CRT system.^84^

### Self-rechargeable leadless devices

One limitation of LPMs is the battery end of life, which inevitably leads to replacement of the LPM with or without extraction of the prior implanted LPM. Self-powered CIEDs may possibly create a solution to this problem. To provide energy to the LPM, kinetic energy originating from the intrinsic cardiac muscle is converted into electric energy. Different models have been tested *in vitro* and *in vivo* (porcine hearts), with success in harvesting energy. More studies are needed to provide evidence on stability and safety of this battery recharging mechanism.^85–87^

### Future directions

Further research should focus on conducting adequately powered long-term prospective randomized trials on LPMs. While pacing-induced cardiomyopathy is associated with chronic RV pacing in TVPM, no long-term studies have been performed to identify the risk of pacing-induced cardiomyopathy in patients with LPMs. Further work is required to assess the change in LVEF due to chronic RV pacing with LPMs.

Furthermore, LPM extraction procedures or the implantation of multiple LPMs simultaneously in the RV needs to be further investigated. In cadaver studies, up to three Micra LPMs could be implanted in the RV septum, without device-to-device interaction.^25^ In humans, there are reports of two simultaneously present LPMs in the RV septum.

Dar *et al*.^88^ reported a success rate of 100% of retrieval of Micra LPMs; 50% of the LPMs were considered late retrievals with median time from implant of 46 days. Also, safe Micra retrieval even after 7 years of implantation has been reported.^89^ However, large trials on safety of extraction of completely endothelialized LPMs are missing. Reddy *et al*.^90^ describe the safe extraction of the Nanostim LPM in 10 of 11 patients with a median of 346 days after LPM implantation. In another study, Nanostim retrieval was successfully extracted in 66 of 73 LPMs with a median time from implant to extraction of 2.9 years. In six patients, the LPM could not be extracted due to inaccessibility of the docking button. Complications were reported in 3% of the patients; in two patients, the docking button was detached from the Nanostim device, and in one patient, an arteriovenous fistula was reported.^9^

### Cost effectiveness

Although LPMs result in less short- and long-term complications attributed to leads and pacemaker (PM) pocket, they are significantly more expensive than TVPM at the time of implantation. Clementy *et al*.^91^ presented the cost of complications related to TVPMs at 3-year follow-up. During follow-up, 5.3% of patients had complications related to TVPMs, mostly consisting of pocket bleeding, mechanical complications related to the lead or generator, and pneumothorax. Device infections occurred infrequently (0.4%), although this was the most expensive complication, with a mean cost of 10.700 euro per infection. In other studies, mean costs of infection were higher, 31.493 euro (Germany) and 30.958 pound per infection (Great Britain) were reported.^92,93^

Certain risk factors, including female sex, low body mass index, renal disease, and recent device infection, were associated with a higher risk of device-related complications. These patients could benefit from LPM implantation, making the LPM more cost effective in higher risk populations (*Table 3*).

## Conclusions

Our review supports the fact that leadless pacing is here to stay. Leadless pacing is a rapid evolving technology suitable for different pacing modalities and various patient populations. Long-term outcomes are promising, and complication rates decreased due to more experience and new developments. With several upcoming leadless technologies and improvements in currently available technologies, the future of pacing is beyond wires.

## Declarations

### Data Availability

No data were generated or analysed for this manuscript.
